# Phytosterol Oleate Ester Replacing Cholesterol to Enhance Lutein Liposome Stability: A Mechanistic Study

**DOI:** 10.3390/foods15030539

**Published:** 2026-02-03

**Authors:** Zimeng Zhao, Pengtao Xu, Zhenchen Luo, Wei Chen, Duoxia Xu, Yanbo Wang, Shaojia Wang

**Affiliations:** Key Laboratory of Geriatric Nutrition and Health (Beijing Technology and Business University), Ministry of Education, School of Food and Health, Beijing Technology and Business University, Beijing 100048, China

**Keywords:** lutein, liposomes, phytosterol oleate ester, stability

## Abstract

Hydrophobic bioactive compounds such as lutein exhibit poor water solubility and are prone to degradation. Liposomal delivery systems can enhance the solubility and physicochemical stability of lutein (LUT). Liposomes are primarily composed of phospholipids and cholesterol. Since phytosterol ester can reduce cholesterol levels and improve the performance of liposomes, this study used phytosterol oleate ester (POE) as a cholesterol substitute in the preparation of liposomes for delivering LUT (LUT-P-Lip). The physicochemical properties, microstructure, storage stability, antioxidant characteristics, and intermolecular interactions of the liposomes at different LUT concentrations were investigated. The results demonstrated that LUT-P-Lip had a size range of 50–100 nm, with intact morphology and uniform distribution. In vitro studies showed that LUT-P-Lip significantly enhanced the storage stability and antioxidant activity of LUT. The analysis of intermolecular interactions revealed that the enhanced stability was mediated by an increased number of hydrogen bonds and modulation of membrane fluidity. In conclusion, replacing cholesterol with POE during liposome formation enhances both the stability and antioxidant activity of the resulting liposomes.

## 1. Introduction

Lutein (LUT) is a typical carotenoid that exhibits strong antioxidant activity arising from the interaction between its polyunsaturated bonds and hydroxyl groups (OH). Miki found that the conjugated polyunsaturated double bonds in the LUT molecule interact with the OH at both ends, enabling efficient free-radical scavenging and conferring potent antioxidant activity [1]. As a precursor of vitamin A, LUT has been demonstrated to be effective in preventing and treating ocular diseases. As a core component of the macular region of the human retina, LUT reduces photodamage by absorbing blue light in the 430–480 nm range and alleviates oxidative stress by scavenging free radicals, exhibiting significant preventive effects against age-related macular degeneration and cataracts [2,3]. However, the structural features that confer LUT’s functionality also reduce its stability. Becerra et al. found that the extended conjugated carbon double bonds in the LUT backbone lead to poor water solubility and increased susceptibility to degradation and isomerization [4], which ultimately leads to low bioavailability in vivo [5]. Techniques such as modification, microencapsulation, nanoemulsification, and liposome technology are commonly employed to overcome LUT’s susceptibility to degradation. Hydroxyl esterification of LUT significantly enhances its stability and improves its tolerance to light- and oxygen-induced degradation. Encapsulation in liposomes further promotes LUT’s water solubility and stability [6,7].

Liposomes were first developed in the 1960s and gained widespread application in drug delivery systems during the 1970s [8], and were introduced into the food industry in the early 1980s. Initially, liposome technology was employed to encapsulate and deliver drugs or enzymes. Encapsulating enzymes (such as amylase) or drugs within liposomes allows the bilayer membrane to shield them from enzymatic degradation, acid–base reactions, or immune system interactions in vivo, significantly enhancing the stability of active ingredients [9]. Structurally, liposomes resemble cell membranes, with phospholipids forming a bilayer through self-assembly to create spherical vesicles. Hydrophilic substances are encapsulated within these vesicles, while hydrophobic substances are embedded within the phospholipid bilayer to protect against oxidation or damage by gastric acid [10]. The addition of sterols to liposomes can enhance membrane stability and fluidity. Iovine et al. demonstrated that incorporating cholesterol during liposome preparation improves stability and fluidity [11]. However, excessive cholesterol intake increases the risk of cardiovascular diseases and hyperlipidemia, among other conditions. Therefore, developing low-risk alternatives to cholesterol is essential for advancing liposome preparation technologies.

Recent studies have demonstrated that phytosterols, such as stigmasterol, soybean sterol, and rapeseed oil sterol, can serve as substitutes for cholesterol in liposome preparation. These compounds not only enhance liposome stability and fluidity but also exhibit antioxidant activity. Incorporation of phytosterols into nanoliposomes has been shown to effectively delay the peroxidation of the liposomal membrane, and after 30 days of storage at 4 °C, the encapsulation efficiency remained largely unchanged, indicating excellent physical stability of the system [12]. Nonetheless, phytosterols present certain limitations, such as low solubility in the oil phase, high melting points, and poor digestibility and absorbability in the human gastrointestinal tract, which consequently restrict their practical applications [13,14]. In contrast, phytosterol esters exhibit higher lipophilicity and lower melting points, thereby significantly enhancing their bioavailability [15]. Commonly used phytosterol esters include butyrate, phytosterol oleate ester (POE), linoleate, and stearate. POE was selected for this study due to its advantages over other esters, including rigidity and fluidity [16], high lipid solubility [17].

In this study, soybean phospholipid was selected as the main membrane material. LUT complex liposomes prepared with either POE or cholesterol were compared in terms of particle size, zeta potential, encapsulation efficiency, and microstructure. Fourier transform infrared spectroscopy, X-ray diffraction, Raman spectroscopy, and differential scanning calorimetry were used to characterize the complex liposomes. The effects of cholesterol and POE on the liposome membrane structure were investigated, and the possible molecular mechanisms underlying these effects were explored.

## 2. Materials and Methods

### 2.1. Materials

LUT (85% purity) was purchased from Chenguang Biotech Co., Ltd. (Quzhou, China), while POE (98.22%) was purchased from Shaanxi Pioneer Biotech Co., Ltd. (Xi’an, China). Soybean phospholipid was purchased from Shanghai Macklin Biochemical Co., Ltd. (Shanghai, China), and cholesterol, potassium ferricyanide, and trichloroacetic acid were purchased from Sinopharm Group (Beijing, China). Ferric chloride was purchased from Macklin Biochemical Co., Ltd. (Beijing, China). The ABTS and 1,1-diphenyl-2-trinitrohydrazine (DPPH) free radical scavenging assay kits were purchased from Beijing Solarbio Science & Technology Co., Ltd. (Beijing, China).

### 2.2. Preparation of LUT Liposomes

LUT-complexed liposomes were prepared using the thin-film hydration ultrasonication method coupled with high-pressure homogenization. Briefly, 1 g of soybean phospholipid and 0.2 g of cholesterol or 0.2 g of POE (*w*/*w* = 5:1) were dissolved in 100 mL of lutein–chloroform solution at lutein concentrations of 0.1, 0.3, 0.5, and 0.7 mg/mL, respectively. The mixture was then vacuum-evaporated at 50 °C and 40 rpm to obtain a homogeneous liposome membrane. Then, 100 mL of phosphate-buffered saline (PBS, 0.2% *w*/*v* Tween 80, 7.4 mM Na_2_HPO_4_, 2.6 mM NaH_2_PO_4_, pH 7.4) was added to hydrate the film for 30 min until its complete separation and dissolution. The membrane was then transferred to a beaker for ultrasonic disruption using an Ultrasonic Homogenizer (JY92-IIN, Ningbo, China) at 400 W for 2 min. Finally, the suspension was homogenized for 3 cycles at 300 bar using a high-pressure microfluidizer (AH-NANO, ATS Engineering, Jiangsu, China), yielding lutein–cholesterol complex liposome (LUT-Lip) and lutein–POE complex liposome (LUT-P-Lip) with LUT concentrations of 0.1, 0.3, 0.5, and 0.7 mg/mL.

### 2.3. Determination of Encapsulation Efficiency of LUT Liposomes

#### 2.3.1. LUT Standard Curve Plotting

This study followed the methodology of Chen et al. with minor modifications [18], employing ultra-performance liquid chromatography (UPLC) for the quantitative determination of LUT. A 10 mg aliquot of lutein standard was precisely weighed, dissolved in anhydrous ethanol, and then diluted to 10 mL with anhydrous ethanol in a brown volumetric flask to prepare a 1 mg/mL stock solution, which was stored at 4 °C. Aliquots of the stock solution were used to prepare LUT-ethanol standard working solutions with different concentrations (25 μg/mL, 50 μg/mL, 75 μg/mL, 100 μg/mL, and 120 μg/mL). A standard curve was plotted with LUT concentration (*x*-axis) against peak area (*y*-axis). The UPLC conditions were as follows: C18 column (50 mm × 2.1 mm, 1.7 μm); mobile phase: acetonitrile: methanol (90:10); flow rate: 0.3 mL/min; column temperature: 25 °C; injection volume: 0.7 μL; detection wavelength: 446 nm. LUT exhibited a linear response over the concentration range of 25–120 μg/mL, with a standard curve described by the equation y = 67,840.82x − 375,059.56 and R^2^ = 0.999.

#### 2.3.2. Calculation of LUT Encapsulation Efficiency

The encapsulation efficiency (EE) of LUT was calculated according to the methods described by Bao [19] and Gan [20], involving centrifugation to separate free LUT from liposomes. To determine the free LUT concentration (C1), 2 mL of the liposome sample was mixed with 6 mL of anhydrous ethanol and centrifuged at 7000 rpm for 20 min. The supernatant was collected, and LUT content was determined using UPLC, as described in Section 2.3.1. The amount of lutein encapsulated in the liposomes was determined by subtracting the free lutein from the total amount used in the preparation. Finally, the EE of LUT was calculated using the following formula (Formula (1)):

EE% = (1 − C_1_/C_0_) × 100% (1)

where EE is the encapsulation efficiency (%); C_1_ is the concentration of free LUT; C_0_ is the total amount of lutein.

### 2.4. Characterization of LUT Liposomes

Liposome particle size, polydispersity index, and zeta potential were measured using a Zetasizer Nano-ZS90 (Malvern Instruments, Worcestershire, UK). Briefly, 0.25 mL of the sample was diluted 200-fold and equilibrated for 120 s at 25 °C prior to measurement. Liposome morphology was observed using a transmission electron microscope (TEM) (JEOL, Tokyo, Japan). Lay a piece of sealing film flat on a glass slide. Place the grids on the sealing film. Using a pipette, apply 10 µL of the sample onto each grid. Allow the sample to adsorb for 10 min. Gently blot away the liquid using filter paper. Then, pipette 10 µL of 2% phosphotungstic acid onto the grid and incubate for 3 min. Carefully blot away the excess staining solution using a small piece of filter paper. Allow the grid to dry for 10 min. Once the grid is completely dry, it is ready for observation under the low-voltage transmission electron microscope. The stability of samples with varying LUT concentrations was analyzed using the LUMiSizer stability analyzer (LUM GmbH, Berlin, Germany) according to the method described by Tai et al. [21], with minor modifications. The experiment was conducted at 25 °C with a sample volume of 0.4 mL, rotation speed of 4000 rpm, and spectral collection every 10 s for a total period of 1 h.

### 2.5. Detection of the Antioxidant Capacity of Liposomes

#### 2.5.1. Calculation of DPPH Free Radical Scavenging Rate

The DPPH free radical scavenging capacity of the samples was determined using a DPPH assay kit (Beijing Solarbio Science & Technology Co., Ltd., Beijing, China). LUT-LIP, LUT-P-LIP, free LUT solution, and free POE solution were each mixed with ethanol solution by vortexing, centrifuged at 10,000 rpm for 10 min at room temperature, and the supernatant was collected and stored on ice for subsequent analysis. After dark incubation at room temperature for 30 min, the absorbance was measured at 515 nm using a microplate reader (MOLECULAR DEVICES SpectraMax i3x, San Jose, CA, USA). The DPPH free radical scavenging rate was calculated according to the following formula:

DPPH free radical scavenging rate (D%) = [(A_Blank_ − (A_measured_ − A_control_)]/A_blank_] × 100% (2)

where A_blank_, A_control_, and A_measured_ represent the absorbance values at 515 nm for the DPPH solution mixed with anhydrous ethanol, the sample solution mixed with anhydrous ethanol, and the sample solution mixed with the DPPH solution, respectively.

#### 2.5.2. Calculation of ABTS Free Radical Scavenging Rate

The ABTS free radical scavenging capacity of the samples was determined using an ABTS assay kit (Beijing Solarbio Science & Technology Co., Ltd., Beijing, China). LUT-LIP, LUT-P-LIP, free LUT solution, and free POE solution were each mixed with ethanol solution by vortexing, centrifuged at 10,000× *g* rpm for 10 min at room temperature, and the supernatant was collected and stored on ice for subsequent analysis. After dark incubation at room temperature for 6 min, the absorbance was measured at 405 nm using an enzyme microplate reader. The ABTS free radical scavenging rate was calculated according to the following formula:

ABTS free radical scavenging rate D% = [(A_Blank_ − (A_measured_ − A_control_)]/A_blank_] × 100% (3)

where A_blank_, A_control_, and A_measured_ represent the absorbance values at 405 nm for the ABTS solution mixed with PBS buffer solution, the sample solution mixed with PBS buffer solution, and the sample solution mixed with the ABTS solution, respectively.

#### 2.5.3. Reducing Capacity Analysis

The reducing capacity of the samples was determined following the method of Tan et al. [22]. Briefly, 1 mL of the sample was pipetted into a test tube, followed by 1 mL of potassium ferricyanide (1% *w*/*w*), and incubated in a 50 °C water bath for 20 min. Then, 1 mL of trichloroacetic acid (10% *w*/*w*) was added, and the mixture was cooled to room temperature and centrifuged at 5000× *g* rpm for 10 min. Thereafter, 2 mL of the supernatant was mixed with 0.4 mL of ferric chloride (0.1% *w*/*w*) and 2 mL of distilled water, and the mixture was allowed to stand for 10 min. The absorbance (A) was measured at 700 nm.

K_3_Fe(CN)_6_ + sample → K_4_Fe(CN)_6_ + sample oxide

K_4_Fe(CN)_6_ + Fe^3+^ → Fe_4_[Fe(CN)_6_]_3_

### 2.6. Intermolecular Interactions

#### 2.6.1. Differential Scanning Calorimetry (DSC)

DSC was conducted following the method of Mao et al. [23], with minor modifications. Approximately 3–10 mg of the sample was sealed in an aluminum crucible and placed on the DSC sample stage, with a reference sample positioned on the opposite side. The sample was heated from 25 to 250 °C at a rate of 10 °C/min under a nitrogen flow of 50 mL/min using the DSC Q200 (TA Instruments Inc., New Castle, DE, USA), and data were recorded throughout the analysis.

#### 2.6.2. X-Ray Diffraction Analysis (XRD)

An adequate amount of sample powder was evenly applied onto the sample stage and gently compacted. The surface was leveled by scraping off excess sample with a glass slide. The sample was then analyzed using an X-ray diffractometer (D8 ADVANCE, Bruker, Bremen, Germany) in step-scanning mode, with the diffraction angle (2θ) set in the range of 5° to 60° and the Cu K radiation at 40 kV and 40 mA for 10 min.

#### 2.6.3. Fourier Transform Infrared Spectroscopy (FTIR)

FTIR analysis of LUT liposomes was performed following the method of Miatmoko et al. [24], with minor modifications. LUT liposomes at four different concentrations were freeze-dried, and 5 mg of the sample was mixed with 100 mg of potassium bromide. After uniform grinding, the mixture was pressed into a pellet at 20 MPa. Subsequently, infrared spectroscopy analysis was performed directly using a Nicolet 6700 spectrometer (Nicolet, Madison, WI, USA), with FTIR detection over the wavenumber range of 4000–400 cm^−1^.

#### 2.6.4. Raman Spectroscopy Detection

Raman spectroscopy was performed following the method described by Cherdchom et al. [25], with minor modifications. A measured amount of powdered sample was placed on a slide, leveled, and positioned on the instrument. The test position was first focused under the light microscope, and spectra were acquired at an excitation wavelength of 785 nm, with a 50 μm aperture slit, and a scanning Raman shift range of 50–3376 cm^−1^.

### 2.7. Statistical Analysis

All measurements were performed on at least three sample preparations and are expressed as the mean ± standard deviation. Statistical data were compared using Duncan’s multiple range test (*p* < 0.05) and one-way analysis of variance in SPSS statistical software (version 24.0; SPSS Inc., Chicago, IL, USA).

## 3. Results and Discussion

### 3.1. Preparation of LUT Liposomes and Variation of Encapsulation Efficiency

**Figure 1:**
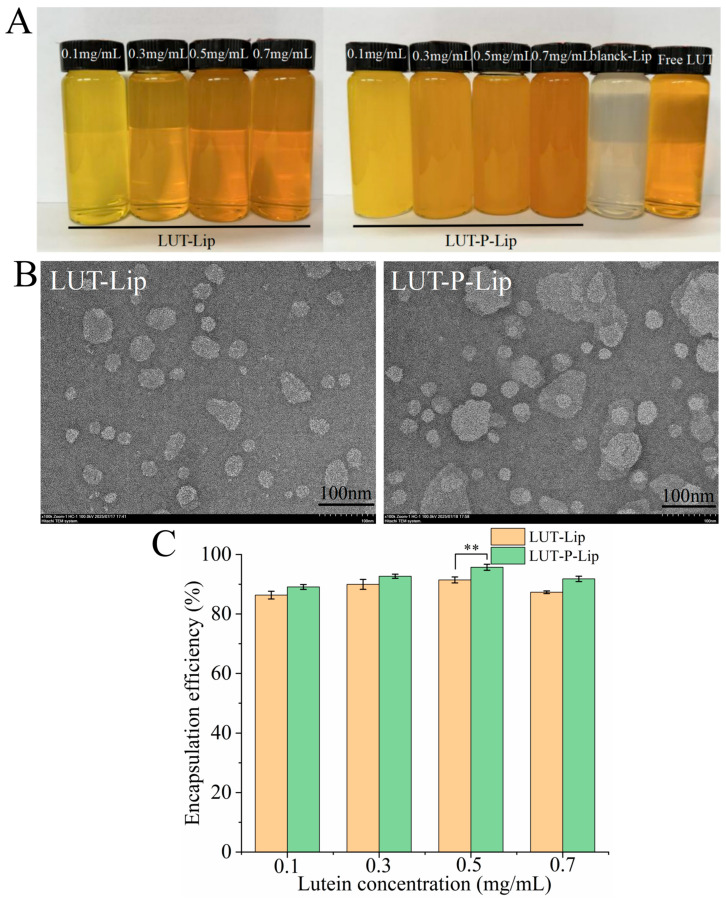
(**A**) The appearance, (**B**) transmission electron microscopy (TEM) images, and (**C**) encapsulation efficiency (EE) of LUT-Lip and LUT-P-Lip at 0.1, 0.3, 0.5, and 0.7 mg/mL. ** *p* < 0.01.

The two types of liposomes prepared in this study exhibited significant differences in appearance. As LUT concentration increased, the liposomes gradually darkened (Figure 1A). Compared to LUT-Lip, LUT-P-Lip showed lower transmittance, and TEM images revealed that LUT-P-Lip was more densely packed, displaying spherical or oval vesicles with monodisperse clusters (Figure 1B). Further analysis of encapsulation efficiency revealed that for both liposome types, EE initially increased with rising LUT concentration and then declined (Figure 1C). Higher EE was observed at LUT concentrations of 0.3 mg/mL and 0.5 mg/mL, with LUT-Lip achieving 89.96% and 91.44 ± 1.00%, respectively. When POE was used as a substitute for cholesterol, EE increased to 95.69 ± 1.02% and 95.69 ± 1.02% at 0.3 mg/mL and 0.5 mg/mL, respectively. Both types of liposomes exhibited a peak in EE because LUT was stably embedded in the vesicles through hydrophobic interactions with the lipid tails. As a result, the EE increased with the rising LUT concentration [26]. However, when the LUT concentration exceeds the capacity of the lipid bilayer, LUT aggregates outside the vesicles or disrupts the bilayer structure, resulting in decreased EE [27]. The increased EE of LUT observed after the substitution with POE might be due to the enhanced compatibility between the oleic acid chain of POE and the hydrophobic structure of LUT, which facilitates more stable incorporation of LUT into the liposomal membrane, reduces leakage, and enhances overall EE [28].

### 3.2. Changes in Liposome Stability

**Figure 2:**
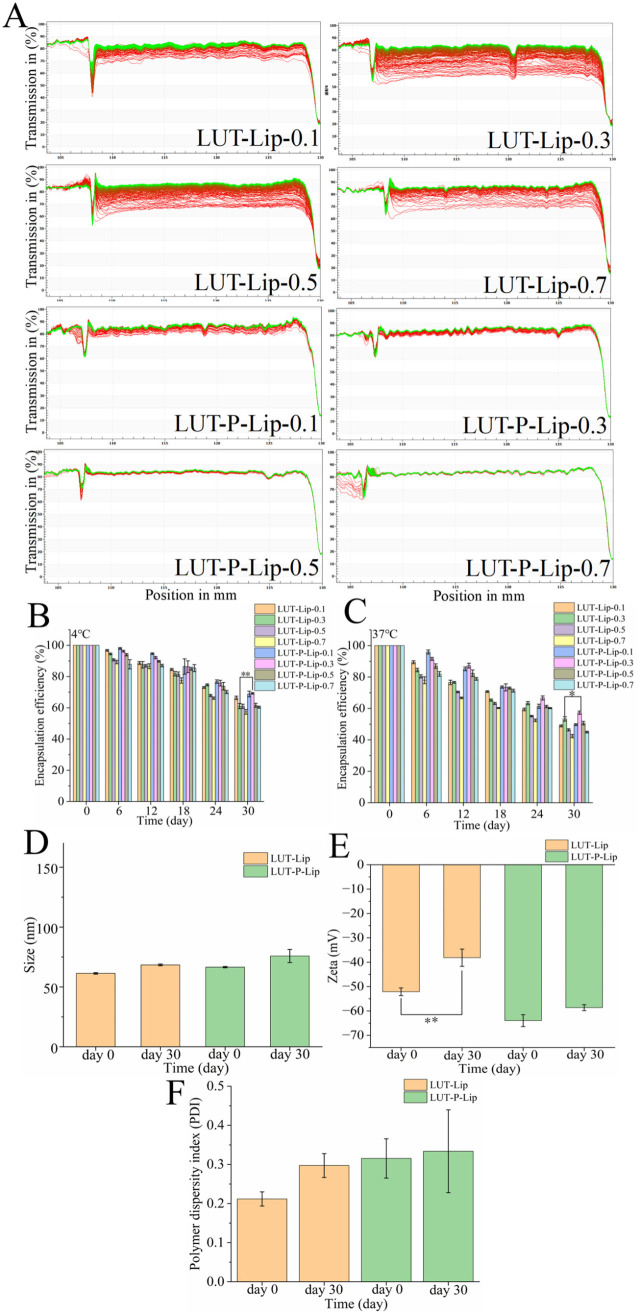
(**A**) LUMiSizer analysis of LUT-Lip and LUT-P-Lip at various LUT concentrations. Storage stability of LUT-Lip and LUT-P-Lip. The encapsulation efficiency at (**B**) 4 °C and (**C**) 37 °C. (**D**) The particle size, (**E**) PDI, and (**F**) zeta potential of both LUT-Lip and LUT-P-Lip were measured at day 0 and day 30, respectively. * *p* < 0.05, ** *p* < 0.01.

To further evaluate the changes in liposome stability, a LUMiSizer was utilized to determine the transmittance variations of liposome samples after centrifugation. In the figure, the red line represents the initial scan at the bottom, while the green line indicates the final scan at the top. Larger changes in light transmittance during centrifugation suggest a faster decline in liposome stability [29]. Compared to LUT-P-LIP, LUT-LIP showed a more pronounced change in transmittance, indicating enhanced stability of LUT-P-LIP (Figure 2A). This may be attributed to POE’s ability to significantly enhance the thermal stability and overall structural integrity of liposomes by increasing alkyl chain ordering in the membrane, reducing polarity, providing antioxidant protection, and inhibiting aggregation [30]. Consequently, POE demonstrates superior stability in LUT delivery systems.

As thermodynamically unstable systems, liposomes are prone to core material leakage [31,32], making the retention rate of the core material a critical indicator for assessing liposome stability. Evaluation of LUT-Lip and LUT-P-Lip at 4 °C (Figure 2B) and 37 °C (Figure 2C) revealed a gradual decline in LUT retention over time, reaching a minimum value at 30 days. Moreover, the retention rate of LUT in liposomes decreased more significantly at 37 °C than at 4 °C. For LUT-P-Lip and LUT-Lip with a concentration of 0.3 mg/mL, the encapsulation efficiency at 4 °C and 37 °C after 30 days of storage dropped from 69.29 ± 0.50% to 57.32 ± 1.01% and 61.14 ± 1.62% to 53.35 ± 1.41%, respectively. The increase in environmental temperature accelerated liposome leakage through two mechanisms: (1) increased oxidation of unsaturated double bonds in phospholipids, thereby disrupting the structure of the liposome membrane; (2) weakening of the hydrophobic interactions and van der Waals forces within the lipid bilayer, enhancing its permeability and fluidity, which facilitates easier leakage of the core material [33,34]. Across all conditions, LUT-P-Lip consistently maintained a higher LUT encapsulation efficiency than LUT-Lip. After 30 days of storage at 4 °C and 37 °C, the encapsulation efficiency of LUT in LUT-Lip (0.3 mg/mL) decreased to 61.14 ± 1.62% and 53.35 ± 1.41%, respectively, while those in LUT-P-Lip were 69.29 ± 0.50% and 57.32 ± 1.01%, respectively. This may be attributed to cholesterol translocation during storage, which disrupts membrane structure. Additionally, due to its enhanced hydrophobicity after esterification, POE becomes stably embedded in the membrane [16]. Furthermore, POE may exert a synergistic antioxidant effect with LUT in liposomes, partially mitigating phospholipid oxidation and thereby improving encapsulation efficiency.

Further analysis of changes in particle size, PDI, and zeta potential of the two liposomes revealed a slight increase in both particle size (Figure 2D) and PDI (Figure 2E) for both liposome formulations after 30 days of storage, but with no significant differences (*p* > 0.05). The absolute values for zeta potential of both LUT-P-Lip and LUT-Lip were greater than 30 mV (Figure 2F). An absolute zeta potential value exceeding 30 mV is widely regarded as a prerequisite for electrostatic stability, as it enhances liposome stability and prolongs their storage duration [35]. This is primarily because the negative charges on the liposome surface generate electrostatic repulsion, thereby reducing liposome aggregation [36]. Compared to Day 1, the zeta potential of LUT-Lip decreased significantly from −52.14 mV to −38.16 mV by Day 30 (** *p* < 0.01). The results of the phytosterol oleate ester fraction further demonstrated that LUT-P-Lip exhibits superior storage stability compared to LUT-Lip.

### 3.3. Changes in the Antioxidant Activity of Liposomes

**Figure 3:**
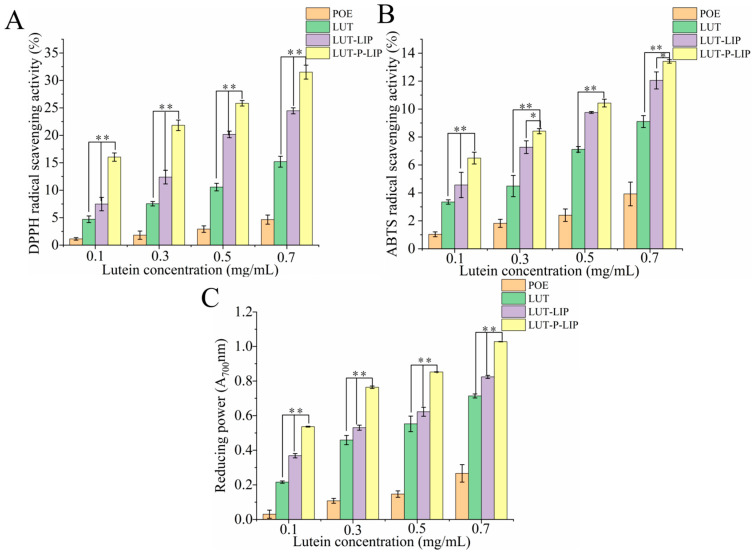
In vitro antioxidant activities: (**A**) DPPH radical scavenging activity; (**B**) ABTS radical scavenging activity; (**C**) Ferric reducing antioxidant power (FRAP). * *p* < 0.05, ** *p* < 0.01.

DPPH, ABTS, and FRAP are three commonly used in vitro methods for detecting antioxidant activity. DPPH and ABTS primarily assess the free radical-scavenging capacity of composite liposomes [37], while FRAP evaluates the total reducing capacity of antioxidants within liposomes [38]. Our study found that LUT encapsulated in liposomes exhibited significantly higher antioxidant activity and reducing capacity than free LUT. Furthermore, substituting cholesterol with POE further enhanced the antioxidant activity and reducing capacity of the liposomes (Figure 3). Using DPPH free radical scavenging rate as an example, the free LUT at a concentration of 0.3 mg/mL showed a DPPH scavenging rate of 7.54 ± 0.40%, while the LUT-Lip liposomes demonstrated a significantly increased DPPH scavenging rate of 12.39 ± 1.25%. When POE was used to substitute cholesterol, the DPPH scavenging rate rose to 21.82 ± 0.96% (Figure 3A). Chen et al. found that liposome-encapsulated astaxanthin exhibited a significantly enhanced antioxidant capacity, with a 35% increase in ABTS free radical scavenging capacity [16].

### 3.4. Intermolecular Interactions

To further explore the mechanisms underlying the improved stability of liposomes after their formation and the molecular basis of these effects, structural changes in the liposomes were investigated using FTIR, X-ray diffraction, Raman spectroscopy, and differential scanning calorimetry.

#### 3.4.1. DSC Analysis

**Figure 4:**
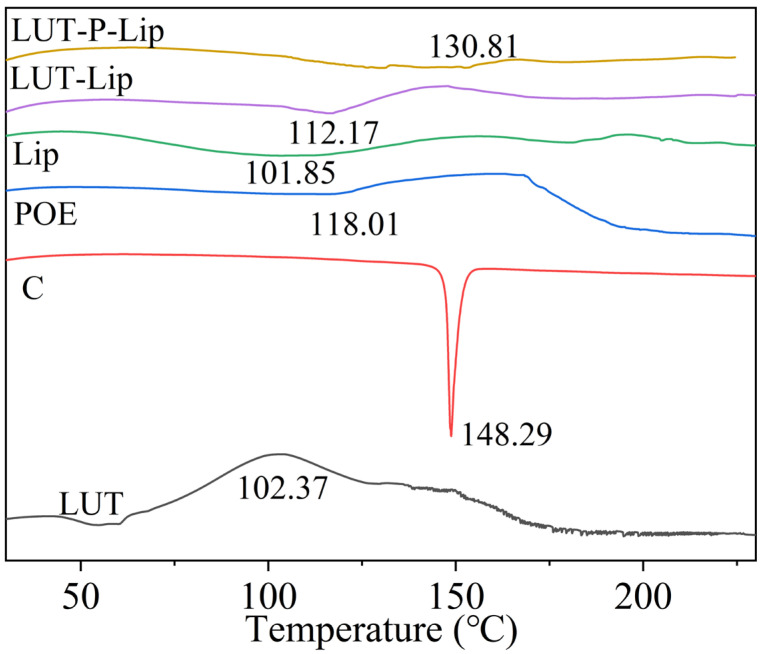
Differential scanning calorimetry (DSC) heating curves of LUT-Lip and LUT-P-Lip.

DSC results demonstrated that free LUT exhibited a phase transition temperature of 102.37 °C (Figure 4). After liposome formation, the originally sharp single peak broadened into an endothermic peak, likely because LUT was encapsulated within the phospholipid bilayer, reducing its crystallinity and enhancing its thermal stability. The first endothermic peak of Lip (Lack of POE and cholesterol) appeared at 101.85 °C. After the formation of LUT-Lip, the melting point increased by approximately 10 °C, which may be attributed to the interaction between the planar rigid steroidal ring of cholesterol and the fatty acid chains of phospholipids, leading to an increased ordered phase of the liposomes. Similar observations have been reported in previous studies, in which incorporating LUT into liposomes led to the formation of ππ interactions between the planar, rigid conjugated double-bond structure of LUT molecules and the unsaturated fatty acid chains of phospholipid molecules, thereby enhancing the ordered phase of the liposomal membrane [39]. After incorporating POE, the phase transition temperature of LUT-P-Lip increased to approximately 130.81 °C, indicating that POE may interact with phosphoric acid, resulting in a more ordered liposome structure and thereby elevating the thermal transition temperature. Similar findings were reported in a previous study, which demonstrated that the variation in phase transition temperature correlated with the arrangement of phosphatidyl chains and the thermal stability of lipid membranes [40]. This implies that LUT-P-Lip exhibits reduced membrane fluidity and a more compact structure at room temperature.

#### 3.4.2. X-Ray Diffraction Analysis

**Figure 5:**
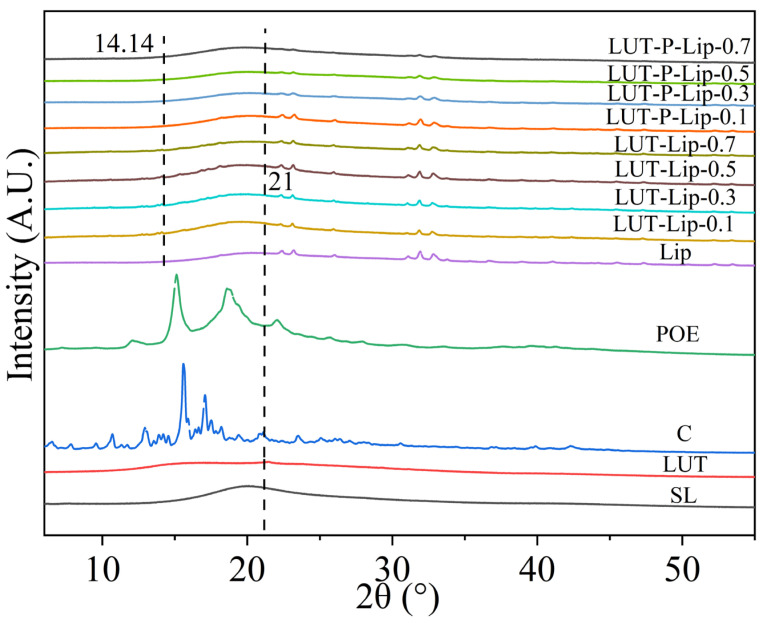
X-ray diffraction (XRD) patterns of LUT-Lip and LUT-P-Lip at varying LUT concentrations.

X-ray diffraction results revealed that the lutein raw material exhibited a characteristic peak at 21°, consistent with its crystalline nature [41]. After lipid preparation, this characteristic peak disappeared and was replaced by amorphous peaks in the 10–20° range, indicating a transition of LUT from a crystalline state to a molecularly dispersed form and successful encapsulation within liposomes. Similarly, phospholipid materials exhibited a broad peak at 16–28° (Figure 5), suggesting that the phospholipids primarily existed in an amorphous, non-crystalline structure. After liposome formation, the positions of the diffraction peaks shifted, and rather than forming a simple superposition of the LUT and phospholipid peaks, they formed a new phase state. Comparing the spectra of the two liposomes, we found that LUT-Lip exhibited a sharp peak at 14°, indicating that cholesterol may crystallize within the phospholipid bilayer, thereby creating localized rigid regions. The disappearance of the sharp peak after incorporating POE indicated that POE was embedded in the phospholipid bilayer in a molecularly dispersed manner, enhancing the overall orderliness of the membrane without generating crystalline rigid zones while maintaining the flexibility and uniformity of the membrane. Hou et al. reported similar findings that after encapsulation in liposomes, POE lost its crystalline features and exhibited an amorphous structure [40], which may have enhanced both the stability and uniformity of liposomes.

#### 3.4.3. FTIR

**Figure 6:**
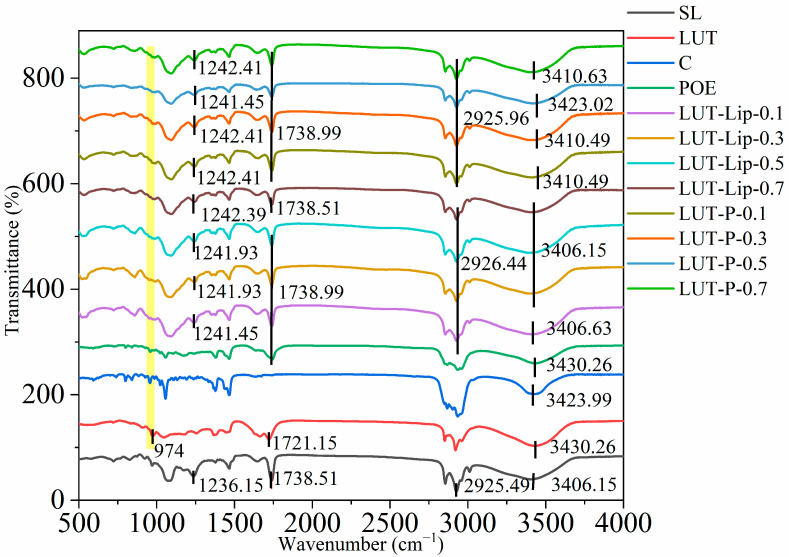
Fourier transform infrared (FTIR) spectra of LUT-Lip and LUT-P-Lip across different LUT concentrations. Yellow vertical bars are used to visually demarcate the spectral regions of interest discussed in the text.

The FTIR results revealed that the vibrational peak of LUT at 974 cm^−1^ disappeared after liposome formation (Figure 6), indicating the successful incorporation of LUT into the phospholipid bilayer. The wavenumber range of 3200–4000 cm^−1^ is the region of hydrogen bond stretching vibration. After liposome formation, the wavenumber of LUT-Lip did not differ significantly from that of the phospholipid raw material. However, the absorption peak underwent a blue shift after the incorporation of POE, with the wavenumber reaching 3423.02 cm^−1^ in the LUT-P-Lip sample with the LUT concentration of 0.5 mg/mL. This suggested that new hydrogen bonds were formed between phospholipids and POE. Junaid Khan et al. [42] reported that after the flavonoids were complexed with phospholipids, the hydrogen bond vibration of their phenolic hydroxyl groups underwent a blue shift from their original positions to 3425 cm^−1^, indicating the formation of new hydrogen bonds. In the LUT-Lip sample, the stretching vibration peak at 2925.49 cm^−1^, which characterizes the CH_2_ stretching vibration of the phospholipid acyl chain, underwent a blue shift to 2926.44 cm^−1^, suggesting that the enhanced bond energy was due to the insertion of LUT and cholesterol into the phospholipid bilayer. Elkholy et al. found that after carotenoids were encapsulated, the symmetric CH_2_ stretching band showed an upward shift in wavenumber [43]. The vibration peaks of the liposomes near wavenumbers of 1242 cm^−1^ and 1738 cm^−1^ represented the phospholipid P=O and C=O bonds, respectively. It was observed that compared to the phospholipid raw material, the absorption peaks of the liposomes underwent a blue shift (particularly at a LUT concentration of 0.5 mg/mL, which underwent a blue shift from 1236.51 cm^−1^ to 1242.41 cm^−1^) after the formation of liposomes, indicating reduced membrane fluidity. This led to more compact molecular packing and stronger intermolecular interactions. Gu J et al. discovered that the vibrations of the phosphate ester (P=O) and ester carbonyl group (C=O) in the phospholipid head both underwent a blue shift after drug encapsulation [44]. These phenomena collectively suggested that the formation of liposomes enhanced membrane rigidity and hindered oxygen permeation due to the rearrangement of hydrogen bonds, thereby significantly improving the storage and oxidative stability of LUT.

#### 3.4.4. Raman Spectroscopy Analysis

**Figure 7:**
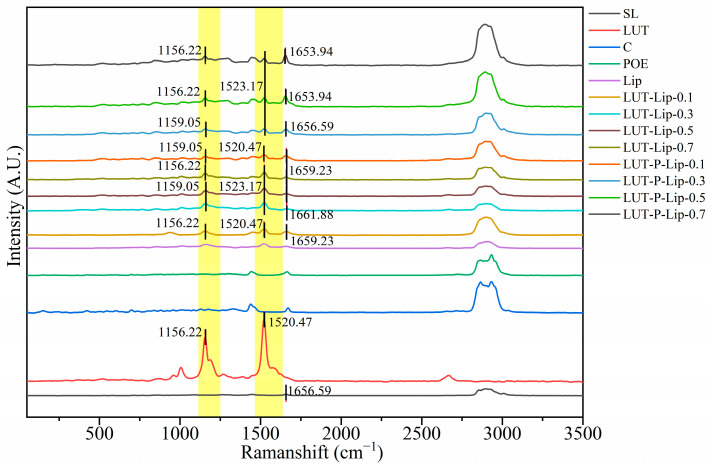
Raman spectra of LUT-Lip and LUT-P-Lip at different LUT concentrations. Yellow vertical bars highlight the spectral regions that are the focus of the discussion.

Raman spectroscopy analysis demonstrated that the C=C stretching vibration of LUT at 1520.47 cm^−1^ underwent a blue shift in peak position and a narrowing in peak width after liposome formation (Figure 7). Specifically, for LUT concentrations of 0.3 mg/mL, 0.5 mg/mL, and 0.7 mg/mL in liposomes, the peak position shifted to 1523.17 cm^−1^. Similarly, the C-C stretching vibration peak at the wavenumber of 1156.22 cm^−1^ either remained unchanged or underwent a blue shift after liposome formation. In LUT-Lip samples, LUT concentrations of 0.3 and 0.5 mg/mL resulted in a blue shift to 1159.05 cm^−1^, whereas in LUT-P-Lip samples, 0.1 mg/mL and 0.3 mg/mL produced the same shift. Sanjay et al. also reported that cyclodextrin’s characteristic peaks shifted to higher wavenumbers after liposome formation [45]. The blue shift of the C=C and C-C peaks and the narrowing of the peak shape may be because, after LUT was embedded in the phospholipid bilayer, the van der Waals forces and hydrophobic interactions may extend the conformation of the LUT molecular chain, resulting in a more ordered arrangement of LUT in the liposomes. The peak at the wavenumber 1656.59 cm^−1^, corresponding to the C=O stretching vibration in the phospholipid raw material, remained unchanged or underwent a blue shift in all samples except for those with LUT concentrations of 0.5 mg/mL and 0.7 mg/mL in LUT-P-Lip, which showed a minor red shift. Marlina et al. also discovered that the original C=O peak at 1656 cm^−1^ shifted to a higher wavenumber when complexed with modified chitosan liposomes [46]. This phenomenon may be attributed to the disruption of intermolecular hydrogen bonds in the phospholipid head molecules caused by the cyclic structures of the inserted LUT and cholesterol. The substitution of cholesterol with POE partially alleviated this disruption.

## 4. Conclusions

In this study, an effective LUT-P-Lip system was developed by substituting cholesterol with POE, resulting in enhanced stability and antioxidant activity of liposomes. Further exploration of its molecular mechanisms revealed that POE alters the interactions between the C=O groups of phospholipid molecules and cholesterol, as well as among the phospholipid molecules themselves. This leads to the formation of a new and more stable hydrogen-bonding network, enhancing the compactness of the membrane structure and strengthening hydrophobic interactions. Consequently, lutein is more stably embedded into the phospholipid bilayer, reducing oxygen molecule leakage and significantly improving the stability and antioxidant activity of the lutein-loaded liposomes.

## Data Availability

The original contributions presented in this study are included in the article. Further inquiries can be directed to the corresponding author.
